# Supplementary material for: Maturation of the Goat Rumen Microbiota Involves Three Stages of Microbial Colonization
Source: Animals (Basel). 2019 Nov 25;9(12):1028. doi: 10.3390/ani9121028 (PMC6941170; doi:10.3390/ani9121028)
Supplement: Supplementary file 1 [file animals-09-01028-s001.pdf]

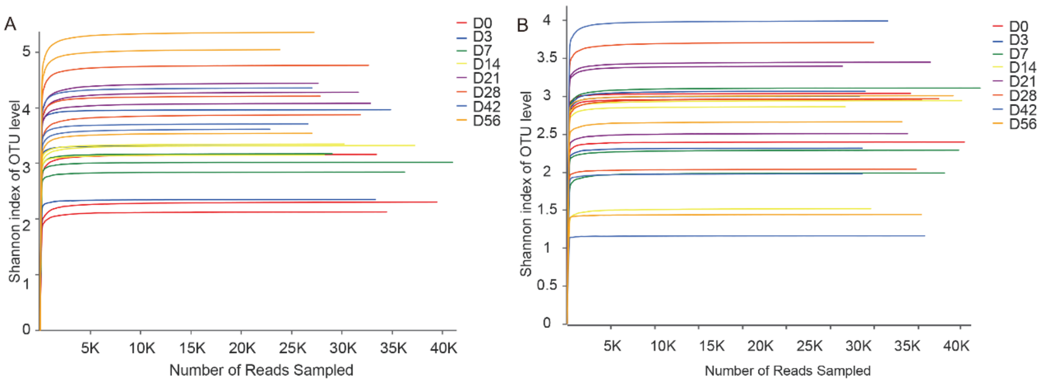

Figure S1. Summary of rarefaction results based on operational taxonomic unit (OTUs) (3% divergence) for each sample. Rarefaction curves are displayed for bacterial samples (**A**). Rarefaction curves for fungal samples (**B**).

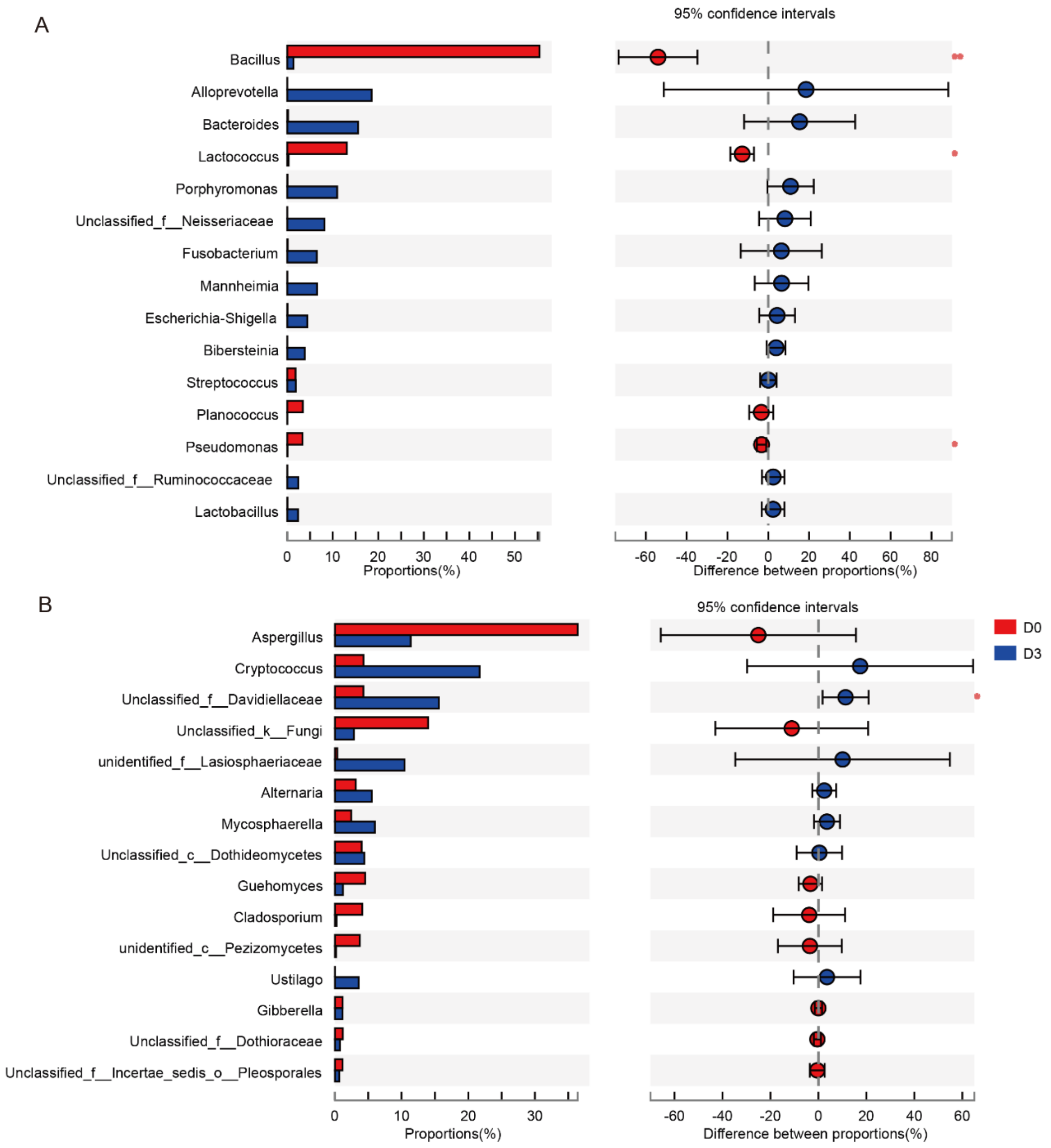

Figure S2. Statistically significance according to the obtained community abundance data at D0 and D3. (**A**) Welch's t-test bar plot on Genus level in bacteria of goats' rumen. (**B**) Welch's t-test bar plot on Genus level in fungi of goats' rumen. * $0.01 < p \le 0.05$, ** $0.001 < p < 0.01$, *** $p \le 0.001$.

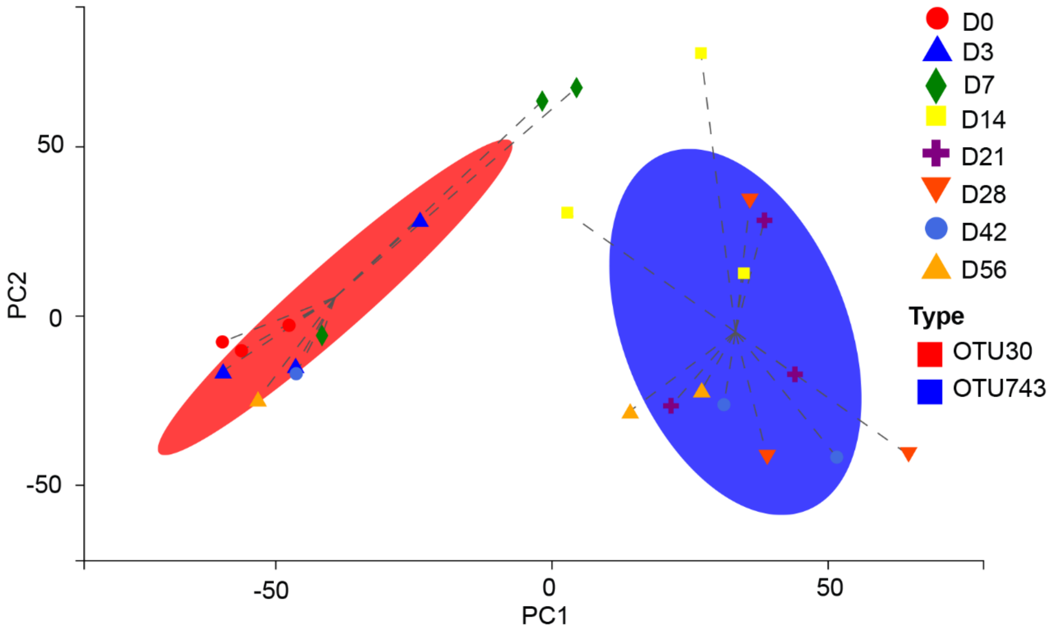

Figure S3. The classification of dominant fungal populations in different samples was studied mainly by statistical clustering.

Table S1. Comparison of the phyla in rumen bacteria of goats (the multiple comparison results were only presented for the phyla, which average relative abundance ≥1% in at least one region). Means in the same row with different superscripts represents a significant difference ($p < 0.05$).

Phylum	D0	D3	D7	D14	D21	D28	D42	D56	SEM	*p* value
Bacteroidetes	6.72^{a}	49.98^{b}	48.91^{b}	64.63^{b}	57.33^{b}	55.38^{b}	68.12^{b}	49.01^{b}	4.06	<0.001
Firmicutes	80.49^{a}	15.12^{c}	16.62^{c}	25.93^{bc}	22.51^{bc}	20.53^{c}	18.08^{c}	37.03^{b}	4.46	<0.001
Proteobacteria	9.68^{bc}	27.16^{a}	16.93^{b}	7.59^{c}	6.33^{c}	3.77^{c}	7.25^{c}	3.53^{c}	1.72	<0.001
Fusobacteria	0.54^{b}	6.56^{b}	16.57^{a}	0.09^{b}	0.01^{b}	0.01^{b}	0.00^{b}	0.00^{b}	1.49	0.019
Spirochaetae	0.02^{b}	0.00^{b}	0.00^{b}	0.48^{b}	1.89^{b}	11.55^{a}	3.01^{b}	5.35^{ab}	1.11	0.086
Lentisphaerae	0.01	0	0	0.27	7.6	5.83	0.33	0.33	0.99	0.318
Fibrobacteres	0.02^{b}	0.00^{b}	0.00^{b}	0.06^{b}	0.39^{ab}	0.57^{ab}	2.41^{a}	1.43^{ab}	0.26	0.191
Actinobacteria	1.96^{a}	0.70^{b}	0.46^{b}	0.16^{b}	0.12^{b}	0.09^{b}	0.11^{b}	0.10^{b}	0.15	0.006
Synergistetes	0.01	0	0.05	0.3	0.81	0.59	0.16	1.53	0.19	0.566
Tenericutes	0	0	0	0.2	1.16	0.21	0.02	0.05	0.11	0.225
Elusimicrobia	0.55^{b}	0.48^{b}	0.45^{b}	0.28^{b}	1.84^{a}	1.48^{b}	0.51^{b}	1.63^{b}	0.09	<0.001

Table S2. Comparison of the predominant genera (average relative abundance ≥5% in at least one GIT region) in rumen bacterial of goats. Means in the same row with different superscripts represents a significant difference (*p < 0.05*).

taxa	D0	D3	D7	D14	D21	D28	D42	D56	SEM	*p* value
Bacillus	55.55[a]	1.40[b]	0.06[b]	0.05[b]	0.02[b]	0.03[b]	0.01[b]	0.02[b]	3.85	<0.001
Pseudomonas	3.31[a]	0.04[b]	0.00[b]	0.01[b]	0.01[b]	0.02[b]	0.00[b]	0.00[b]	0.23	<0.001
Planococcus	3.39[a]	0.02[b]	0.02[b]	0.01[b]	0.03[b]	0.01[b]	0.00[b]	0.00[b]	0.27	0.001
Alloprevotella	0.01	18.49	0.21	7.26	1.5	4.93	0.36	0.21	2.17	0.404
Prevotellaceae_UCG-003	0.20[b]	0.00[b]	2.75[ab]	3.25[ab]	8.69[a]	3.03[ab]	3.91[ab]	2.94[ab]	0.86	0.308
Treponema_2	0.02[b]	0.00[b]	0.00[b]	0.24[b]	1.31[b]	11.13[a]	2.98[ab]	5.26[ab]	1.09	0.09
Prevotellaceae_unclassified	0.40[b]	0.02[b]	0.21[b]	0.27[b]	0.12[b]	4.31[b]	10.98[a]	1.49[b]	0.91	0.06
Ruminococcaceae_unclassified	0.00[d]	2.81[bc]	5.36[a]	4.91[ab]	0.78[cd]	0.68[cd]	0.03[d]	0.12[d]	0.49	<0.001
Rikenellaceae_unclassified	0.00[b]	0.00[b]	0.00[b]	0.00[b]	6.01[a]	4.36[ab]	0.06[b]	0.01[b]	0.66	0.062
Fusobacterium	0.09[b]	6.55[b]	16.55[a]	0.08[b]	0.01[b]	0.01[b]	0.00[b]	0.00[b]	1.5	0.019
Porphyromonas	0.04[c]	11.08[ab]	17.34[a]	5.23[bc]	0.02[c]	0.17[c]	0.00[c]	0.00[c]	1.47	<0.001
Bacteroidales_S24-7_group_norank	0.11	0	0	0.1	0.22	0.19	3.44	11.88	1.31	0.27
Lentisphaerae_RFP12_gut_group_norank	0	0	0	0.21	6.81	5.38	0.24	0.21	0.98	0.457
Bacteroides	0.19[b]	15.69[ab]	25.30[a]	15.10[ab]	0.41[b]	1.47[b]	0.47[b]	0.59[b]	2.37	0.007
Bacteroidales_BS11_gut_group_norank	0.02[b]	0.00[b]	0.00[b]	14.26[ab]	20.63[a]	19.88[a]	0.24[b]	1.31[b]	2.48	0.039

Table S3. The CowPi predicted functions of the rumen bacterial microbiota throughout the goat populations.

ID	D0_1	D0_2	D0_3	D3_1	D3_2	D3_3	D7_1	D7_2	D7_3	D14_1	D14_2	D14_3	D21_1	D21_2	D21_3	D28_1	D28_2	D28_3	D42_1	D42_2	D42_3	D56_1	D56_2	D56_3
Transporters	6.27	6.21	6.13	5.26	4.38	3.41	3.61	4.90	4.95	3.87	3.52	3.41	3.68	3.94	3.58	4.36	3.29	3.87	3.46	3.34	3.58	4.08	4.18	3.80
Two-component system	3.98	3.86	3.97	3.19	3.20	3.26	3.26	2.88	2.94	3.18	2.91	3.37	3.14	3.50	3.26	3.68	2.92	3.39	2.98	3.11	3.25	3.49	3.09	3.35
ABC transporters	3.28	3.23	3.40	3.48	2.80	2.06	2.15	3.39	3.50	2.52	2.33	2.08	2.31	2.38	2.23	2.78	2.07	2.46	2.04	1.94	2.06	2.51	2.44	2.30
Transcription factors	2.99	2.94	2.84	2.07	1.79	1.33	1.51	1.75	1.65	1.92	1.66	1.59	1.65	1.81	1.72	1.73	1.55	1.69	1.40	1.25	1.50	1.75	1.58	1.56
DNA repair and recombination proteins	2.45	2.48	2.51	3.10	3.09	3.27	3.20	3.32	3.33	3.12	3.28	3.21	3.21	3.08	3.19	3.15	3.22	3.17	3.13	3.22	3.23	3.18	3.46	3.21
Purine metabolism	2.15	2.18	2.19	2.49	2.39	2.65	2.48	2.60	2.58	2.57	2.62	2.52	2.60	2.47	2.58	2.52	2.63	2.55	2.68	2.72	2.67	2.68	2.72	2.61
Other ion-coupled transporters	2.13	2.12	1.99	1.61	1.56	1.42	1.46	1.58	1.57	1.61	1.55	1.50	1.42	1.46	1.49	1.39	1.52	1.42	1.35	1.26	1.37	1.33	1.47	1.34
Ribosome	2.04	2.06	2.09	2.44	2.34	2.59	2.43	2.54	2.53	2.51	2.74	2.48	2.73	2.50	2.69	2.57	2.78	2.69	2.58	2.67	2.57	2.57	2.65	2.64
Peptidases	2.01	2.04	1.99	1.97	1.97	2.29	2.08	2.16	2.17	2.09	2.23	2.18	2.16	2.15	2.21	2.13	2.27	2.17	2.19	2.30	2.21	2.16	2.21	2.22
Chromosome	1.75	1.74	1.74	1.87	1.71	1.84	1.80	1.81	1.79	1.73	1.83	1.78	1.74	1.66	1.68	1.70	1.74	1.66	1.72	1.74	1.67	1.75	1.98	1.71
General function prediction only	1.74	1.73	1.74	1.85	1.85	1.86	1.84	1.86	1.89	1.74	1.78	1.78	1.89	1.74	1.77	1.62	1.71	1.73	1.75	1.79	1.76	1.73	1.92	1.71
Ribosome Biogenesis	1.60	1.60	1.63	1.99	1.99	2.02	1.96	2.03	2.01	1.93	1.97	1.96	2.02	1.89	1.94	2.09	2.00	1.99	2.09	2.14	2.01	2.10	2.06	2.13
Sporulation	1.53	1.54	1.28	0.31	0.14	0.34	0.20	0.26	0.21	0.28	0.34	0.27	0.25	0.35	0.32	0.30	0.26	0.29	0.23	0.27	0.26	0.33	0.37	0.31
Bacterial motility proteins	1.52	1.38	1.52	0.72	0.45	0.37	0.36	0.37	0.44	0.45	0.56	0.37	0.91	0.61	0.66	1.53	0.47	1.08	0.56	0.69	0.95	1.17	1.14	0.94
Pyrimidine metabolism	1.49	1.53	1.53	1.89	1.85	2.11	1.93	2.07	2.04	2.07	2.22	2.03	2.18	2.05	2.21	2.02	2.22	2.16	2.19	2.22	2.12	2.16	2.20	2.14
Amino acid related enzymes	1.46	1.49	1.50	1.66	1.63	1.83	1.80	1.74	1.73	1.87	2.03	1.86	1.98	1.89	1.99	1.89	2.05	1.96	1.94	1.98	1.95	1.92	1.95	1.96
Glycolysis / Gluconeogenesis	1.25	1.24	1.17	0.89	0.83	0.76	0.77	0.80	0.77	0.79	0.78	0.75	0.82	0.82	0.80	0.73	0.75	0.77	0.76	0.74	0.75	0.73	0.75	0.75
Amino sugar and nucleotide sugar metabolism	1.24	1.27	1.23	1.27	1.38	1.47	1.45	1.31	1.31	1.37	1.43	1.46	1.51	1.51	1.53	1.47	1.44	1.53	1.53	1.50	1.58	1.45	1.55	1.51
Pyruvate metabolism	1.24	1.23	1.18	1.03	0.96	0.87	0.94	1.03	1.03	0.94	0.81	0.88	0.81	0.82	0.78	0.81	0.76	0.76	0.83	0.76	0.79	0.87	0.73	0.80
Arginine and proline metabolism	1.19	1.20	1.19	1.01	0.97	1.17	1.09	0.96	0.92	1.17	1.27	1.12	1.12	1.16	1.18	1.10	1.15	1.12	1.17	1.20	1.19	1.15	1.12	1.16
Phosphotransferase system (PTS)	1.11	1.11	0.96	0.38	0.33	0.23	0.13	0.28	0.30	0.14	0.09	0.09	0.20	0.18	0.13	0.21	0.12	0.16	0.25	0.13	0.31	0.19	0.39	0.18
Protein kinases	1.07	1.06	1.08	0.96	1.05	1.50	1.20	0.87	0.89	1.04	0.81	1.23	0.85	1.16	1.00	0.98	0.91	0.94	1.09	1.07	1.10	1.11	0.84	1.04
Starch and sucrose metabolism	1.01	1.03	0.99	1.00	1.22	1.51	1.56	1.03	1.02	1.32	1.20	1.55	1.28	1.47	1.33	1.35	1.30	1.30	1.40	1.47	1.39	1.27	1.01	1.36

Chaperones and folding catalysts	1.00	1.00	1.04	1.29	1.28	1.41	1.26	1.19	1.18	1.15	1.20	1.19	1.15	1.14	1.14	1.19	1.21	1.16	1.39	1.31	1.30	1.18	1.19	1.25
Carbon fixation pathways in prokaryotes	0.99	1.00	0.99	1.06	1.02	1.02	1.12	1.22	1.22	1.06	1.00	1.12	0.98	0.97	0.95	0.95	0.99	0.93	0.98	1.02	1.02	1.05	1.15	0.97
Methane metabolism	0.99	1.01	0.98	1.18	1.18	1.15	1.26	1.33	1.31	1.33	1.39	1.29	1.28	1.27	1.30	1.23	1.28	1.26	1.07	1.08	1.00	1.10	1.11	1.17
Secretion system	0.98	0.94	0.99	0.92	0.82	0.76	0.66	0.77	0.77	0.69	0.84	0.62	0.95	0.72	0.77	0.86	0.79	0.85	0.74	0.74	0.74	0.80	0.86	0.77
Aminoacyl-tRNA biosynthesis	0.97	0.99	1.01	1.23	1.20	1.33	1.27	1.37	1.38	1.33	1.46	1.32	1.41	1.33	1.43	1.36	1.45	1.41	1.34	1.40	1.39	1.42	1.48	1.40
Transcription machinery	0.94	0.96	0.97	1.09	1.27	1.35	1.24	1.10	1.04	1.28	1.39	1.35	1.30	1.30	1.37	1.23	1.45	1.30	1.38	1.45	1.30	1.27	1.12	1.37
Cysteine and methionine metabolism	0.94	0.95	0.92	0.90	0.91	0.94	1.05	0.95	0.96	1.09	1.19	1.04	1.07	1.07	1.06	1.05	1.15	1.03	0.96	1.01	0.97	0.99	0.97	1.04
Alanine, aspartate and glutamate metabolism	0.94	0.96	0.96	0.99	1.09	1.18	1.17	1.07	1.07	1.21	1.27	1.21	1.29	1.23	1.29	1.24	1.33	1.28	1.25	1.33	1.24	1.24	1.12	1.29
Lipid biosynthesis proteins	0.94	0.94	0.94	0.86	0.84	0.68	0.87	0.78	0.76	0.95	0.90	0.89	0.95	0.96	0.98	0.92	1.01	0.99	0.83	0.89	0.79	0.86	0.82	0.93
Oxidative phosphorylation	0.90	0.91	0.91	0.92	0.92	1.01	1.09	0.84	0.87	0.92	0.99	0.99	0.85	0.91	0.89	0.82	0.95	0.86	0.95	0.97	0.95	0.85	0.83	0.89
Pentose phosphate pathway	0.89	0.89	0.87	0.81	0.86	0.76	0.82	0.75	0.76	0.80	0.73	0.78	0.76	0.81	0.78	0.73	0.81	0.75	0.78	0.73	0.80	0.69	0.70	0.73
Glycine, serine and threonine metabolism	0.88	0.89	0.89	0.89	0.84	0.94	0.90	0.84	0.80	1.05	1.22	1.00	1.12	1.09	1.21	1.00	1.15	1.15	0.94	0.97	0.92	0.95	0.87	1.01
Nitrogen metabolism	0.87	0.87	0.85	0.77	0.87	0.70	0.85	0.82	0.85	0.78	0.72	0.82	0.71	0.76	0.75	0.69	0.72	0.72	0.73	0.72	0.76	0.73	0.73	0.71
Others	0.83	0.81	0.82	0.69	0.73	0.53	0.69	0.61	0.61	0.72	0.68	0.71	0.66	0.68	0.67	0.59	0.58	0.62	0.64	0.66	0.67	0.65	0.66	0.66
DNA replication proteins	0.82	0.84	0.87	1.21	1.13	1.28	1.21	1.22	1.22	1.22	1.25	1.22	1.28	1.13	1.23	1.24	1.27	1.23	1.30	1.32	1.27	1.30	1.37	1.28
Fructose and mannose metabolism	0.82	0.84	0.77	0.60	0.79	0.77	0.66	0.57	0.57	0.61	0.63	0.64	0.63	0.73	0.70	0.66	0.71	0.69	0.93	0.83	0.87	0.64	0.67	0.75
Butanoate metabolism	0.79	0.79	0.78	0.72	0.67	0.64	0.67	0.92	0.89	0.74	0.62	0.70	0.61	0.64	0.59	0.54	0.57	0.57	0.59	0.61	0.64	0.70	0.75	0.58
Bacterial chemotaxis	0.78	0.70	0.81	0.44	0.32	0.28	0.22	0.36	0.44	0.30	0.36	0.25	0.61	0.42	0.44	1.01	0.33	0.71	0.42	0.46	0.61	0.71	0.70	0.62
Propanoate metabolism	0.77	0.77	0.73	0.58	0.52	0.49	0.52	0.66	0.65	0.54	0.46	0.51	0.49	0.48	0.44	0.47	0.42	0.44	0.48	0.49	0.48	0.56	0.52	0.48
Lysine biosynthesis	0.77	0.78	0.77	0.83	0.78	0.93	0.91	0.85	0.82	1.01	1.10	0.97	1.01	0.99	1.05	1.01	1.05	1.03	0.99	1.03	1.03	1.00	0.95	1.03
Flagellar assembly	0.76	0.70	0.74	0.22	0.13	0.03	0.06	0.08	0.10	0.14	0.18	0.12	0.30	0.20	0.24	0.66	0.15	0.45	0.19	0.28	0.38	0.47	0.44	0.39
Peptidoglycan biosynthesis	0.74	0.75	0.74	0.85	0.80	0.86	0.81	0.90	0.88	0.87	0.99	0.86	0.95	0.89	0.93	0.93	0.92	0.94	0.90	0.94	0.95	0.94	0.96	0.95
Pentose and glucuronate interconversions	0.72	0.74	0.68	0.61	0.78	0.93	0.74	0.56	0.55	0.69	0.66	0.72	0.59	0.76	0.67	0.65	0.67	0.64	0.95	0.81	0.94	0.59	0.53	0.70
Phenylalanine, tyrosine and tryptophan biosynthesis	0.71	0.72	0.74	0.81	0.88	1.05	0.97	0.80	0.80	1.01	1.12	0.99	1.05	1.05	1.11	1.04	1.14	1.09	1.07	1.06	1.07	1.02	0.96	1.07
Fatty acid biosynthesis	0.70	0.69	0.68	0.64	0.62	0.43	0.60	0.60	0.60	0.66	0.61	0.59	0.65	0.61	0.64	0.57	0.62	0.64	0.55	0.58	0.49	0.60	0.50	0.59

Citrate cycle (TCA cycle)	0.69	0.69	0.68	0.72	0.67	0.64	0.69	0.72	0.68	0.67	0.59	0.71	0.59	0.58	0.60	0.50	0.60	0.55	0.58	0.56	0.58	0.57	0.73	0.53
Valine, leucine and isoleucine biosynthesis	0.67	0.67	0.68	0.62	0.58	0.71	0.61	0.50	0.46	0.69	0.78	0.63	0.75	0.72	0.74	0.68	0.76	0.74	0.74	0.70	0.70	0.69	0.56	0.71
Translation proteins	0.66	0.66	0.70	1.02	1.02	0.94	0.96	1.06	1.07	0.90	0.89	0.92	0.94	0.87	0.88	0.87	0.89	0.87	0.98	0.93	0.90	0.94	0.99	0.91
Glyoxylate and dicarboxylate metabolism	0.63	0.63	0.63	0.58	0.58	0.57	0.53	0.55	0.54	0.61	0.56	0.55	0.57	0.58	0.59	0.53	0.60	0.59	0.53	0.44	0.50	0.51	0.44	0.51
Cell cycle - Caulobacter	0.61	0.61	0.63	0.67	0.64	0.70	0.68	0.63	0.62	0.66	0.71	0.71	0.71	0.67	0.70	0.80	0.69	0.73	0.76	0.80	0.76	0.77	0.79	0.79
Pantothenate and CoA biosynthesis	0.60	0.61	0.60	0.59	0.58	0.73	0.67	0.63	0.61	0.62	0.67	0.65	0.65	0.66	0.63	0.67	0.67	0.65	0.70	0.72	0.75	0.71	0.74	0.68
Homologous recombination	0.59	0.60	0.63	0.93	0.89	0.98	0.89	1.03	1.03	0.95	1.04	0.93	1.03	0.91	1.00	0.98	1.04	1.00	0.99	1.02	1.00	1.03	1.11	1.02
Mismatch repair	0.57	0.58	0.60	0.84	0.83	0.77	0.86	0.96	0.99	0.84	0.85	0.85	0.86	0.81	0.84	0.85	0.83	0.83	0.75	0.79	0.81	0.84	0.95	0.82
Glycerophospholipid metabolism	0.55	0.55	0.54	0.62	0.62	0.52	0.56	0.60	0.63	0.57	0.48	0.54	0.55	0.50	0.51	0.52	0.55	0.51	0.57	0.54	0.56	0.54	0.57	0.55
Energy metabolism	0.53	0.54	0.55	0.74	0.83	0.77	0.80	0.93	0.92	0.86	0.85	0.87	0.83	0.81	0.85	0.74	0.87	0.81	0.83	0.81	0.79	0.80	0.85	0.78
Galactose metabolism	0.52	0.53	0.52	0.51	0.62	0.73	0.65	0.50	0.48	0.57	0.57	0.65	0.56	0.65	0.61	0.61	0.58	0.60	0.62	0.65	0.62	0.55	0.48	0.61
Thiamine metabolism	0.52	0.52	0.47	0.38	0.36	0.43	0.40	0.44	0.44	0.36	0.26	0.37	0.34	0.36	0.31	0.34	0.26	0.31	0.42	0.42	0.47	0.42	0.49	0.39
Membrane and intracellular structural molecules	0.51	0.51	0.54	0.78	0.68	0.77	0.62	0.64	0.64	0.60	0.66	0.57	0.62	0.59	0.60	0.62	0.62	0.61	0.75	0.62	0.65	0.60	0.58	0.62
Porphyrin and chlorophyll metabolism	0.51	0.51	0.52	0.71	0.78	0.67	0.79	1.02	1.09	0.52	0.39	0.69	0.41	0.56	0.41	0.48	0.41	0.42	0.61	0.56	0.62	0.63	0.87	0.50
Protein folding and associated processing	0.50	0.49	0.54	0.64	0.65	0.63	0.57	0.64	0.66	0.49	0.39	0.50	0.44	0.48	0.43	0.55	0.42	0.47	0.60	0.55	0.56	0.56	0.59	0.54
Germination	0.49	0.49	0.40	0.07	0.00	0.01	0.02	0.03	0.01	0.04	0.09	0.03	0.05	0.10	0.08	0.08	0.05	0.06	0.01	0.02	0.01	0.03	0.02	0.04
RNA degradation	0.48	0.48	0.50	0.62	0.65	0.60	0.72	0.64	0.65	0.60	0.56	0.67	0.57	0.57	0.56	0.63	0.57	0.56	0.61	0.65	0.62	0.58	0.59	0.61
One carbon pool by folate	0.48	0.49	0.50	0.61	0.67	0.73	0.78	0.69	0.69	0.73	0.92	0.79	0.74	0.77	0.76	0.76	0.82	0.75	0.77	0.82	0.77	0.74	0.73	0.77
Sulfur metabolism	0.48	0.47	0.47	0.45	0.47	0.42	0.51	0.39	0.38	0.48	0.42	0.45	0.43	0.45	0.41	0.42	0.43	0.41	0.40	0.39	0.38	0.39	0.37	0.42
Bacterial secretion system	0.47	0.45	0.49	0.58	0.59	0.53	0.48	0.55	0.57	0.47	0.58	0.45	0.62	0.49	0.52	0.47	0.57	0.53	0.51	0.51	0.47	0.46	0.52	0.49
Folate biosynthesis	0.46	0.45	0.45	0.43	0.48	0.34	0.39	0.41	0.44	0.36	0.36	0.37	0.38	0.35	0.35	0.30	0.38	0.33	0.35	0.31	0.32	0.30	0.32	0.32
Protein export	0.46	0.46	0.46	0.41	0.41	0.41	0.38	0.40	0.40	0.42	0.48	0.39	0.45	0.43	0.45	0.40	0.45	0.44	0.43	0.41	0.43	0.44	0.44	0.43
Signal transduction mechanisms	0.46	0.47	0.44	0.40	0.37	0.59	0.35	0.39	0.39	0.36	0.39	0.38	0.44	0.41	0.45	0.64	0.41	0.54	0.60	0.62	0.58	0.54	0.52	0.62
Selenocompound metabolism	0.46	0.46	0.44	0.42	0.44	0.38	0.47	0.47	0.46	0.47	0.54	0.45	0.44	0.47	0.42	0.49	0.48	0.42	0.48	0.48	0.48	0.47	0.45	0.49
Terpenoid backbone biosynthesis	0.45	0.46	0.46	0.51	0.55	0.63	0.58	0.58	0.58	0.58	0.67	0.60	0.63	0.61	0.64	0.59	0.65	0.63	0.60	0.61	0.62	0.60	0.57	0.61

Glycosyltransferases	0.44	0.45	0.46	0.48	0.59	0.52	0.62	0.47	0.47	0.52	0.51	0.57	0.49	0.54	0.50	0.48	0.51	0.48	0.62	0.60	0.65	0.49	0.49	0.53
Valine, leucine and isoleucine degradation	0.43	0.42	0.42	0.28	0.21	0.20	0.22	0.29	0.25	0.28	0.24	0.24	0.20	0.24	0.23	0.18	0.20	0.21	0.17	0.17	0.17	0.24	0.22	0.18
Histidine metabolism	0.41	0.43	0.45	0.57	0.62	0.64	0.72	0.61	0.62	0.73	0.75	0.73	0.68	0.69	0.72	0.62	0.71	0.68	0.67	0.68	0.64	0.67	0.60	0.66
DNA replication	0.41	0.42	0.45	0.65	0.61	0.67	0.65	0.70	0.70	0.71	0.73	0.69	0.73	0.65	0.72	0.72	0.76	0.71	0.68	0.71	0.72	0.74	0.76	0.72
Sulfur relay system	0.40	0.38	0.37	0.29	0.26	0.21	0.20	0.20	0.22	0.19	0.14	0.17	0.20	0.15	0.14	0.15	0.13	0.14	0.20	0.13	0.15	0.17	0.15	0.15
Carbon fixation in photosynthetic organisms	0.39	0.39	0.41	0.53	0.55	0.59	0.55	0.53	0.54	0.54	0.57	0.56	0.59	0.57	0.59	0.52	0.64	0.56	0.61	0.59	0.59	0.53	0.55	0.56
Fatty acid metabolism	0.38	0.38	0.39	0.32	0.28	0.22	0.28	0.36	0.35	0.36	0.28	0.32	0.29	0.32	0.32	0.28	0.36	0.32	0.22	0.24	0.22	0.28	0.28	0.28
Nicotinate and nicotinamide metabolism	0.37	0.39	0.40	0.52	0.54	0.68	0.64	0.58	0.56	0.60	0.61	0.66	0.61	0.61	0.61	0.60	0.63	0.61	0.67	0.67	0.69	0.64	0.68	0.63
Glycerolipid metabolism	0.35	0.35	0.33	0.28	0.29	0.24	0.20	0.25	0.24	0.25	0.18	0.20	0.24	0.26	0.23	0.26	0.22	0.24	0.22	0.20	0.24	0.24	0.30	0.25
Biosynthesis of unsaturated fatty acids	0.35	0.34	0.33	0.22	0.19	0.12	0.18	0.16	0.15	0.21	0.16	0.18	0.17	0.19	0.17	0.16	0.16	0.18	0.19	0.18	0.15	0.18	0.15	0.17
Translation factors	0.35	0.35	0.38	0.54	0.56	0.56	0.55	0.60	0.59	0.56	0.62	0.54	0.59	0.56	0.58	0.55	0.57	0.58	0.60	0.60	0.59	0.57	0.60	0.58
Ubiquinone and other terpenoid-quinone biosynthesis	0.33	0.34	0.31	0.23	0.26	0.27	0.22	0.19	0.20	0.18	0.27	0.20	0.23	0.21	0.22	0.23	0.25	0.22	0.34	0.31	0.30	0.21	0.24	0.26

Table S4. Comparison of the phyla in rumen fungal of goats (the multiple comparisons results are only presented for the phyla which average relative abundance ≥1% in at least one region). Means in the same row with different superscripts represents a significant difference ($p < 0.05$).

phylum	D0	D3	D7	D14	D21	D28	D42	D56	SEM	*p* value
Ascomycota	74.11	68.14	38.91	45.85	23.42	32.85	26.28	29.99	5.93	0.24
Neocallimastigomycota	0.06^{a}	0.01^{a}	1.74^{a}	16.30^{ab}	67.42^{b}	62.75^{b}	63.96^{b}	56.98^{ab}	8.14	0.029
Basidiomycota	11.71	29.04	14.15	25.68	5.47	2.02	9.41	12.95	3.66	0.639
Fungi_unclassified	13.82^{b}	2.76^{b}	44.24^{a}	10.32^{b}	3.59^{b}	2.30^{b}	0.31^{b}	0.07^{b}	3.91	0.047
Chytridiomycota	0.00^{b}	0.00^{b}	0.31^{b}	1.84^{a}	0.04^{b}	0.04^{b}	0.00^{b}	0.00^{b}	0.18	0.125
Zygomycota	0.14^{b}	0.05^{b}	0.64^{a}	0.01^{b}	0.01^{b}	0.03^{b}	0.04^{b}	0.00^{b}	0.06	0.17
Others	0.15	0	0	0	0.04	0.01	0	0	0.02	0.513

Table S5. Comparison of the predominant genera (average relative abundance ≥5% in at least one GIT region) in rumen fungal of goats. Means in the same row with different superscripts represents a significant difference ($p < 0.05$).

taxa	D0	D3	D7	D14	D21	D28	D42	D56	SEM	*p* value
Cryptococcus	4.26	21.73	5.9	23.78	3.86	1.39	7.4	11.37	3.32	0.638
Fungi_unclassified	13.82^{b}	2.76^{b}	44.24^{a}	10.32^{b}	3.59^{b}	2.31^{b}	0.31^{b}	0.07^{b}	3.92	0.047
Neocallimastigaceae_unclassified	0.03^{c}	0.00^{c}	1.13^{c}	0.00^{c}	19.55^{ab}	4.68^{c}	12.30^{ab}	34.74^{a}	3.53	0.1
Orpinomyces	0.00^{b}	0.00^{b}	0.46^{b}	0.00^{b}	4.79^{b}	34.43^{a}	31.21^{a}	0.55^{b}	3.85	0.034
Caecomyces	0.01^{b}	0.00^{b}	0.14^{b}	12.78^{ab}	20.97^{a}	23.02^{a}	0.53^{b}	2.32^{b}	2.55	0.028
Aspergillus	36.03^{a}	11.02^{b}	1.09^{b}	0.60^{b}	0.38^{b}	0.30^{b}	3.37^{b}	0.76^{b}	2.82	0.001
Neocallimastix	0.01	0	0	0	19.15	0.46	19.75	5.79	3.13	0.493
Cladosporium	8.42^{ab}	15.90^{a}	3.80^{b}	0.35^{b}	1.52^{b}	1.34^{b}	3.07^{b}	6.64^{b}	1.28	0.016
Lasiosphaeriaceae_unidentified	0.37	10.44	0.47	15.37	7.7	6.26	0.01	0.01	2.03	0.443
Alternaria	3.08	5.58	1.64	0.28	1.13	1.46	3.75	6.95	0.83	0.49
Piromyces	0.01^{b}	0.00^{b}	0.00^{b}	3.52^{ab}	2.97^{ab}	0.15^{b}	0.17^{b}	13.59^{a}	1.38	0.16
Davidiella	2.59^{b}	6.41^{a}	0.36^{b}	0.02^{b}	1.06^{b}	1.71^{b}	2.34^{b}	2.09^{b}	0.52	0.045
Ascomycota_unclassified	0.31	0.48	12.78	0.28	0.08	0.36	0.17	0.65	1.53	0.437
Epicoccum	3.95^{ab}	4.40^{a}	2.09^{abc}	0.02^{c}	0.32^{c}	0.22^{c}	0.27^{c}	0.70^{bc}	0.46	0.039

Table S6. Ingredients and nutrients of the experimental diets %.

Ingredients	Content (%)	Nutrient level (DM)	Content (%)
Corn	23.3	Dry matter	89.09
Soybean meal	13.75	Digestible energy (MJ/kg）	8.5
Rice straw	20.4	Crude protein	10
Straw	39.6	Crude fat	2.02
Limestone	0.55	Crude fiber	17.28
$Ca (HCO_3)_2$	0.9	NFE	49.2
NaCl	0.5	Ash	8.36
Premix	1	Calcium	0.7
Total	100	Phosphorus	0.4

Note, the main nutrients of alfalfa include: crude protein 18.1%, lysine 1.76%, methionine + cystine 0.46%, calcium 1.5%, phosphorus 0.49%.
